# The quantification of 3D-trabecular architecture of the fourth cervical vertebra using CT osteoabsorptiometry and micro-CT

**DOI:** 10.1186/s13018-023-03760-2

**Published:** 2023-04-12

**Authors:** Amélie Poilliot, Max Hans-Peter Gay-Dujak, Magdalena Müller-Gerbl

**Affiliations:** Department of Biomedicine, Musculoskeletal Research, Institute of Anatomy, University of Basel, Basel, Switzerland

**Keywords:** Cancellous bone, Cervical vertebra, Computed tomography osteoabsorptiometry, Endplate mineralisation, Micro-computed tomography, Trabecular architecture

## Abstract

**Background:**

Bone functional adaptation rationalises the inhomogeneous morphology found in bone. By means of computed tomography osteoabsorptiometry and micro-computed tomography, the mineralisation of the subchondral endplates and trabecular microstructure of vertebral bodies can be assessed to visualise the chronic loading conditions bone endures over time. In this study, we determined cancellous and compartment-specific trabecular architecture in the cervical vertebra to aid with successful integration of orthopaedic implants.

**Methods:**

We examined the micro-computed tomography scans of seven prospectively healthy C4 vertebrae, evaluated their microstructure parameters (bone volume fraction (BV/TV), bone surface density (BS/BV), trabecular thickness (Tb.Th), trabecular separation (Tb.Sp), trabecular number per volume (Tb.N), connectivity density (Conn.D), structure model index (SMI), and degree of anisotropy (DA), and compared the trabecular architecture in twelve predefined volumes of interest: the cranial and caudal 0–10%, 10–15%, and 25–50% in both the ventral and dorsal half. Using computed tomography osteoabsorptiometry, the subchondral bone mineralisation of the subchondral endplates of nine C4 vertebrae was also evaluated.

**Results:**

Highest mineralisation is located dorsally at the endplates. Tb.Sp and Tb.N were the only two parameters that displayed significant differences in averaged values of VOI. Nonetheless, distinct, consistent ventral–dorsal modulations were seen in matched sample ventral–dorsal comparison in the BV/TV, BS/BV, and SMI overall levels, as well as in Tb.Th in the three caudal levels. To simplify, the vertebra was split into ventral–cranial, dorsal–cranial, ventral–caudal, and dorsal–caudal equal quarters. The ventral quarters display lower BV/TV, respectively, higher BS/BV and SMI than their sample paired dorsal quarters. The ventral–cranial quarter shows the lowest BV/TV and the highest BS/BV and SMI, describing spacious cancellous bone with rod-like trabeculae. In contrast, the dorsal–caudal quarter exhibits the highest BV/TV and Tb.Th and the lowest BS/BV and SMI, illustrating thicker, denser, and more plate-like trabeculae. The dorsal–cranial and ventral–caudal quarters are comparable and represent intermediate characteristics.

**Conclusions:**

CT-OAM and µCT demonstrate the interdependence of compact and trabecular bone in response to long-term loading conditions. Results show highest mineralisation in the dorso-caudal part of the C4 vertebra. Recommended placement of orthopaedic implants should be positioned dorsally with screws anchored in the dorsal–caudal region.

**Supplementary Information:**

The online version contains supplementary material available at 10.1186/s13018-023-03760-2.

## Introduction

Subaxial cervical spine instrumentation surgeries are widely employed procedures, with various techniques for an even wider range of indications [1–3] Although these operations restore stability and cervical lordosis, diverse complications can occur. These include (but are not limited to): screw breakage and pull-out, screw-plate migration, and cage subsidence. In part, these complications lead to reoperations, and in cases where the complications are not necessarily dire presently, they remain undesired to avoid possible late predicaments [4, 5].

Bone perceives mechanical strain and adapts its three-dimensional microstructure to optimally resist the recognised type of load. This modification is known as bone functional adaptation and rationalises the inhomogeneous morphology found in trabecular and compact bone. Computed tomography osteoabsorptiometry (CT-OAM) has already allowed the visualisation of the chronic loading conditions that the cervical spine endplates endure over time [6]. However, in-depth regional trabecular assessment is lacking. Determination of regional bony architecture should lead to the assessment of biomechanical strain on these regions and subsequently assist in surgical planning regarding screw and cage placement, as well as predicting possible fracture sites. Moreover, this information about the trabecular microstructure of healthy cervical vertebrae could assist with our understanding of the progression of metabolic skeletal disorders like osteoporosis. In fact, the literature on the cancellous architecture of the cervical vertebra is sparse and often focuses on the comparison between spinal levels rather than the composition of individual vertebrae [7, 8].

This study aimed to describe the microarchitecture of region-specific cancellous bone in the fourth cervical vertebra and pair results with those of the subchondral endplates using CT-OAM. To determine the chronic loading situation at the cervical spine, the C4 vertebra was selected as it is centrally placed, thus representing the overall loading conditions of the cervical vertebrae in the central cervical spine. For this purpose, we examined the subchondral bone density distribution of the superior and inferior endplates and micro-computed tomography (µCT) scans and of seven prospectively healthy C4 vertebrae and evaluated their microstructure parameters.

## Material and methods

### Specimens

Seven formalin-fixed human C4 cervical vertebrae (four males, three females; age 38–58 years, mean 49.6 years) from the anatomical institute Ludwigs-Maximilians-Universität in Munich were analysed. Scan quality was improved by removing soft tissue prior to analysis. No apparent pathologies could be distinguished radiographically except mild osteoporosis, a normal attribute for the age group. The samples were acquired from individuals who donated their bodies to research at the Ludwig Maximilian University of Munich.

### Compliance with ethical standards

All procedures performed were in accordance with the ethical standards of the institutional and national research committee and with the 1964 Helsinki Declaration and its later amendments or comparable ethical standards.

### CT-osteoabsorptiometry of the subchondral endplates

Data sets for CT-OAM were derived from conventional CT (Siemens Somatom S4). This cohort consisted of seven CT scans of C4 vertebrae from the Anatomical institute Ludwigs-Maximilians-Universität in Munich. Slice thickness averaged 2 mm. CT-OAM was evaluated using a specific image analysis program (ANALYZE) as done previously [9]. The superior and inferior endplates of the C4 vertebra were manually segmented within the CT datasets before the data were false colour-coded and superimposed on the 3-dimensionally reconstructed C4 for anatomical localisation of the bone mineral density creating a colour-map or ‘densitogram’. The maximum intensity projection revealed the HU of each pixel to a depth of 3 mm and threshold values were chosen to be ≤ 200 to ≥ 1200 HU.

### Micro-computed tomography for evaluation of trabecular architecture

**Fig. 1:**
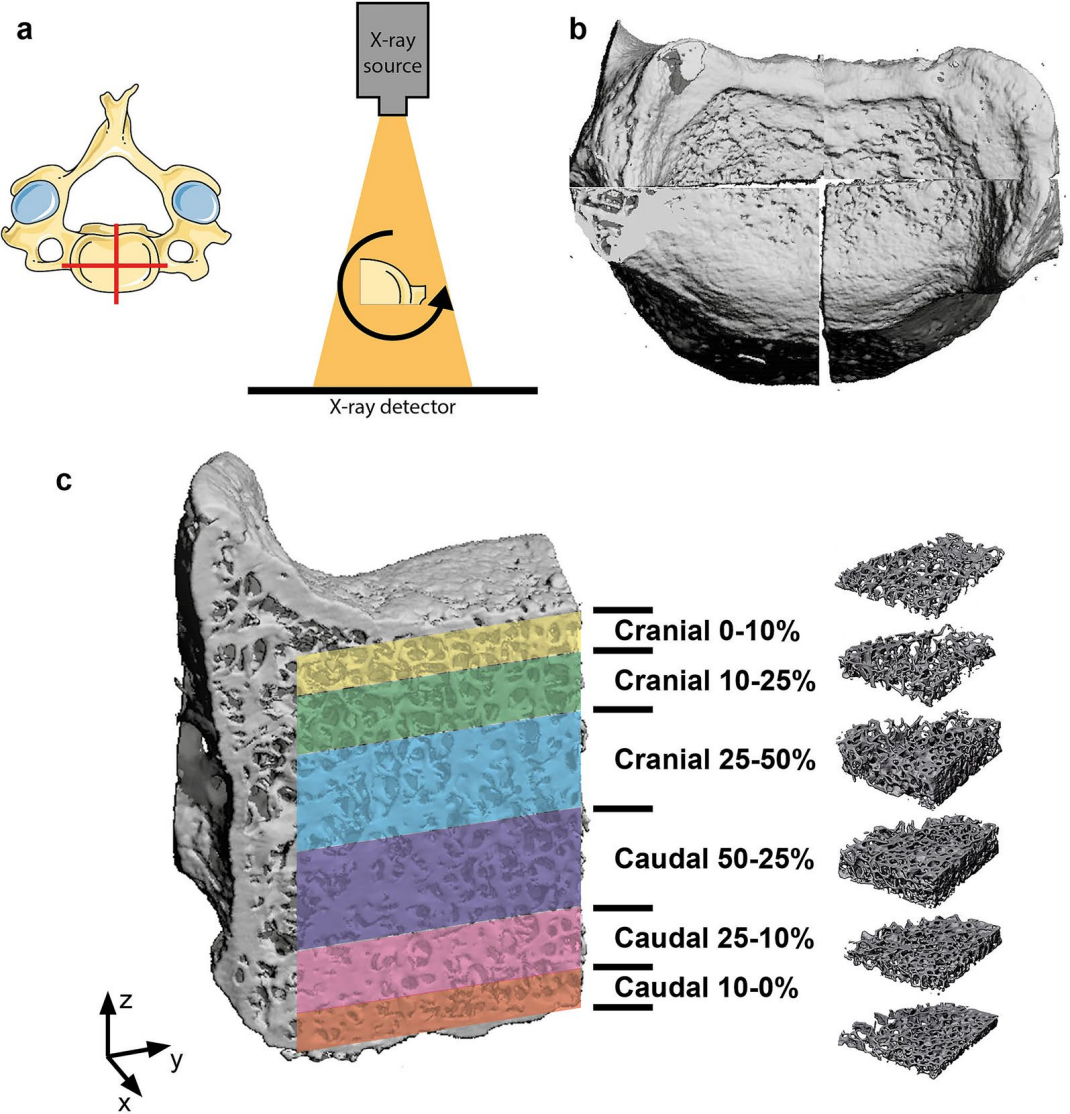
**a** The 4th cervical vertebra was quartered by sagittal and frontal cuts in the median of the vertebral body. The individual quarters were then scanned under 360° rotation and acquisition. **b** Scans were reconstructed in 3D **c** volumes of interest were isolated relative to the absolute height of the trabecular bone by a percentage calculation of 10%, 15% and 25% of the absolute height both caudally and cranially

First, samples were quartered in the centre by frontal and sagittal slices, as to fit within the nanotom. The respective samples were subsequently scanned using a phoenix nanotom® m system (Wunstorf, Germany) (Fig. 1a). Three-dimensional data were acquired using an acceleration voltage of 140 kV, beam current of 60 µA, and a 0.1 mm aluminium filter to reduce beam hardening. Each scan included 2100 projections from 360 degrees, accounting for a 32 µm isotropic voxel size. Three-dimensional reconstruction was conducted according to the Feldkamp algorithm [10] (Fig. 1b).

Volumes of interest (VOI) for trabecular analysis were manually isolated with the help of analysing software VG-Studio® MAX 2.1 (Heidelberg, Germany). Six VOIs were defined relative to the two endplates of each vertebra. These are the cranial and caudal 0–10%, 10–25%, and 25–50% calculated relative to the size of the complete VOI (Fig. 1c). Trabecular architecture measurements were performed by the Skyscan software CT-analyser® (Bruker. Microct, Belgium) on all 168 VOIs (seven samples × four quarters × six VOIs).

Parameters of trabecular bone architecture were selected in accordance with those described in the literature as recommendations of bone analysis [11–13]:

- bone volume fraction (BV/TV [%]) is the fraction of bone volume in regards to the total volume
- bone surface density (BS/BV [1/mm]) is the bone surface present within the total volume
- trabecular thickness (Tb.Th [mm]) is the mean thickness of the trabeculae
- trabecular separation (Tb.Sp [mm]) is the trabecular spacing as a parameter of the mean distance between each single trabecula.
- trabecular number (Tb.N [1/mm]), is the number of trabeculae per volume as a structural parameter.
- connectivity density (Conn.D [1/mm^3^]), indicates the number of connections between trabeculae per unit volume
- structure model index (SMI [dimensionless]), quantifies the characteristic form of a three-dimensionally described structure in terms of the amount of plate- and rod-composing parts of the structure. 0 represents plate-like trabeculae, 3 rod-like trabeculae, and values between the two correlate to the volume ratio between rods and plates [12].
- degree of anisotropy (DA [dimensionless]), quantifies how highly oriented substructures are within a volume of trabecular bone.

### Statistical analysis

Plots and statistical comparisons of all parameters for every VOI were executed using GraphPad Prism (version 8, San Diego, CA, USA). Gaussian distribution was first assessed using a Shapiro–Wilk test. As the data were normally distributed, a two-way ANOVA test with Sidak's multiple comparison post-test was performed. Statistical significance was considered with *p* < 0.05. Mean values were reported ± standard deviation.

## Results

### CT-osteoabsorptiometry revealed increased dorsal mineralisation in the superior and inferior endplates

**Fig. 2:**
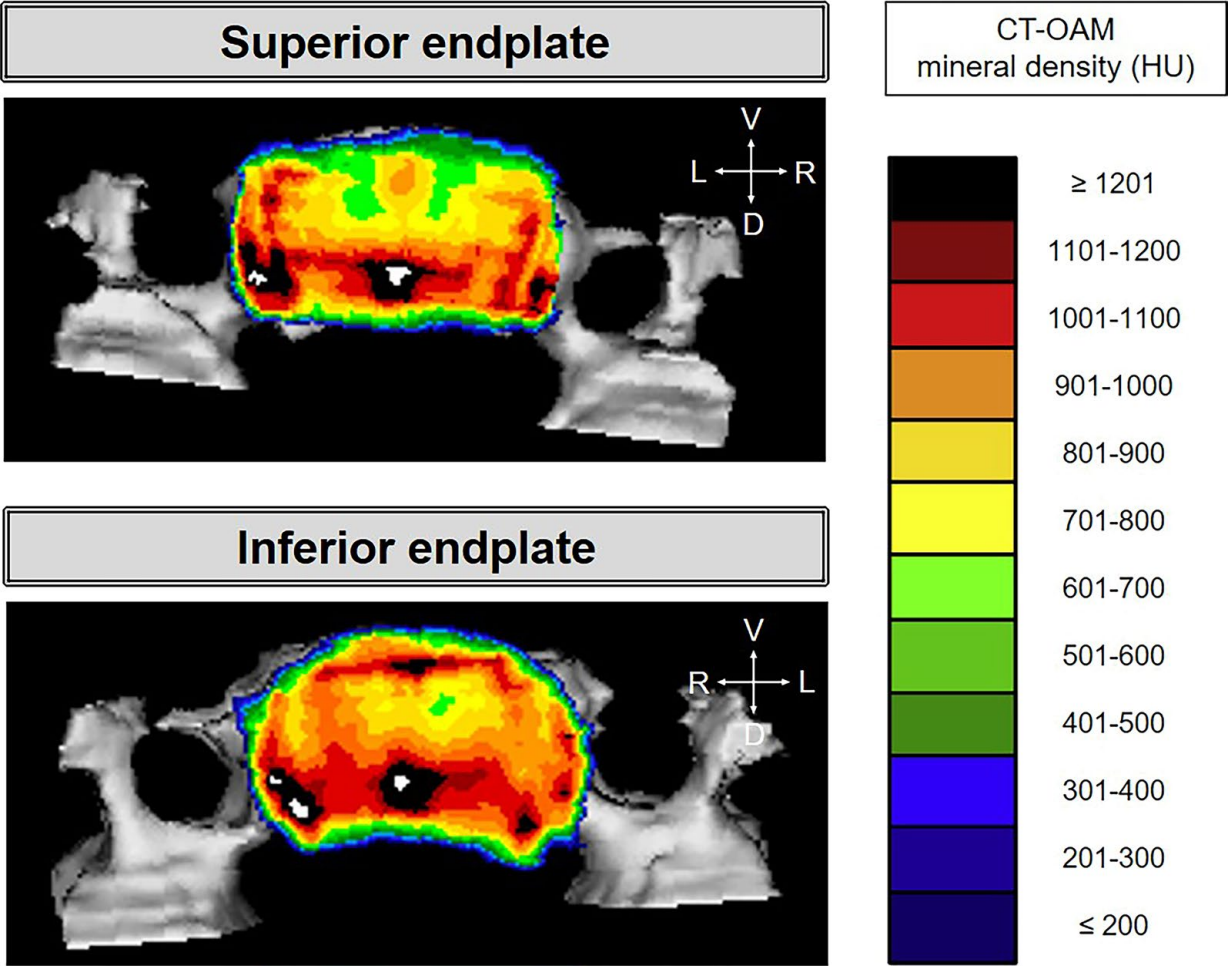
Superior and inferior endplates of one C4 vertebrae. D: dorsal, L: left, R: right, V: ventral

Superior and inferior endplates reveal higher dorsal mineralisation with often lateral dorsal localised mineralisation maxima localised around the borders (Fig. 2).

### Trabecular network parameters are consistent between right and left segments

Each quarter of the C4 vertebra was divided into six VOI layers. No significant differences were seen in the trabecular network parameters between right and left VOIs of the same level in the two ventral quarters, or in the two dorsal quarters (Additional file 1: Figure S1, Additional file 2: Figure S2). Therefore, the right and left VOIs of the same level were averaged to form a cranial and a dorsal VOI on each vertebra.

### The dorsal segments showed higher bone volume than the corresponding ventral regions with an increase towards the core

**Fig. 3:**
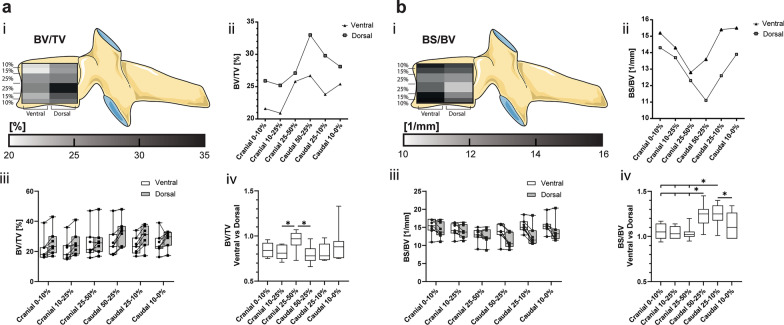
**a** Bone volume fraction defined as bone volume (BV) versus total volume (TV) and **b**) bone surface density defined as bone surface (BS) versus BV are represented in (i) a heatmap corresponding to their cranial–caudal and ventral–dorsal location, (ii) a scatter plot of the mean values of the ventral and dorsal VOI, (iii) a box-and-whisker plot with the individual values for ventral (circle) and dorsal (square) VOI connected by a line (-), and (iv) a box-and-whisker plot of the sample matched ventral–dorsal ratio. (*p < 0.05)

At first glance, the averaged BV/TVs were similar between all the VOIs, ranging from 20.9 ± 7.6% (ventral cranial 10%-25%) to 33 ± 8.6% (dorsal 50–25%) (Fig. 3a). However, when comparing the individual values of the dorsal VOI to their vertebra-matched ventral VOI, lower values were consistently found in the ventral VOI, with the exception of the cranial 25–50% segment. In this segment, the BV/TV of the matched ventral and dorsal VOI were practically even (Fig. 3a).

### The ventral segment showed higher bone surface density than the corresponding dorsal regions with an increase towards the endplates

Similarly, the averaged BS/BV were in similar ranges in all VOIs ranging from 11.1 ± 2 mm^−1^ (dorsal caudal 50–25%) to 15.5 ± 2.2 mm^−1^ (ventral caudal 10–0%). Matched ventral–dorsal VOI ratios show distinct differences between the central and peripheral regions. Comparing the dorsal and ventral VOI of the individual cranial segments reveals no detectable differences but there is a trend towards the ventral regions having a higher BS/BV than their corresponding dorsal regions (Fig. 3b).

### Higher trabecular thickness was seen dorsally compared to the ventral region, with an increase towards the core

**Fig. 4:**
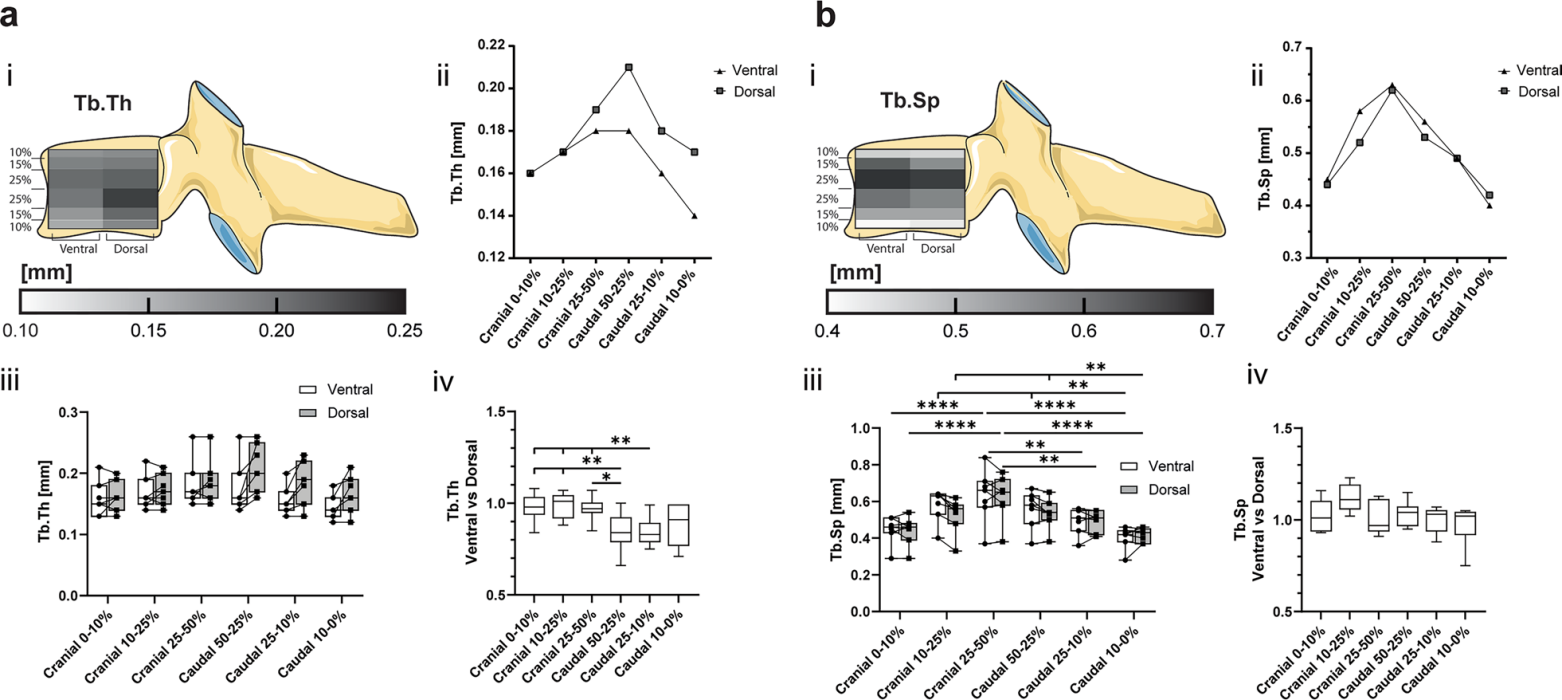
**a** Trabecular thickness (Tb.Th) and **b** trabecular separation (Tb.Sp) are represented in (i) a heatmap corresponding to their cranial–caudal and ventral–dorsal location, (ii) a scatter plot of the mean values of the ventral and dorsal VOI, (iii) a box-and-whisker plot with the individual values for ventral (circle) and dorsal (square) VOI connected by a line (-), and (iv) a box-and-whisker plot of the sample matched ventral–dorsal ratio. (*p < 0.05)

There were no significant differences in the average ventral vs dorsal Tb.Th. The matched ventral–dorsal ratio of the cranial VOI implies a consistent trabecular thickness in the cranial 50% of the vertebra. However, the equivalent ratios of the caudal segments were found to inversely correlate to the bone surface density. The ventral trabeculae are 15% thinner in the caudal 50–25% and 25–10% than in their matched dorsal VOI; however, unchanged in the caudal 10–0% (Fig. 4a).

### Trabecular separation increased towards the core, whilst trabecular number increased towards the extremities

**Fig. 5:**
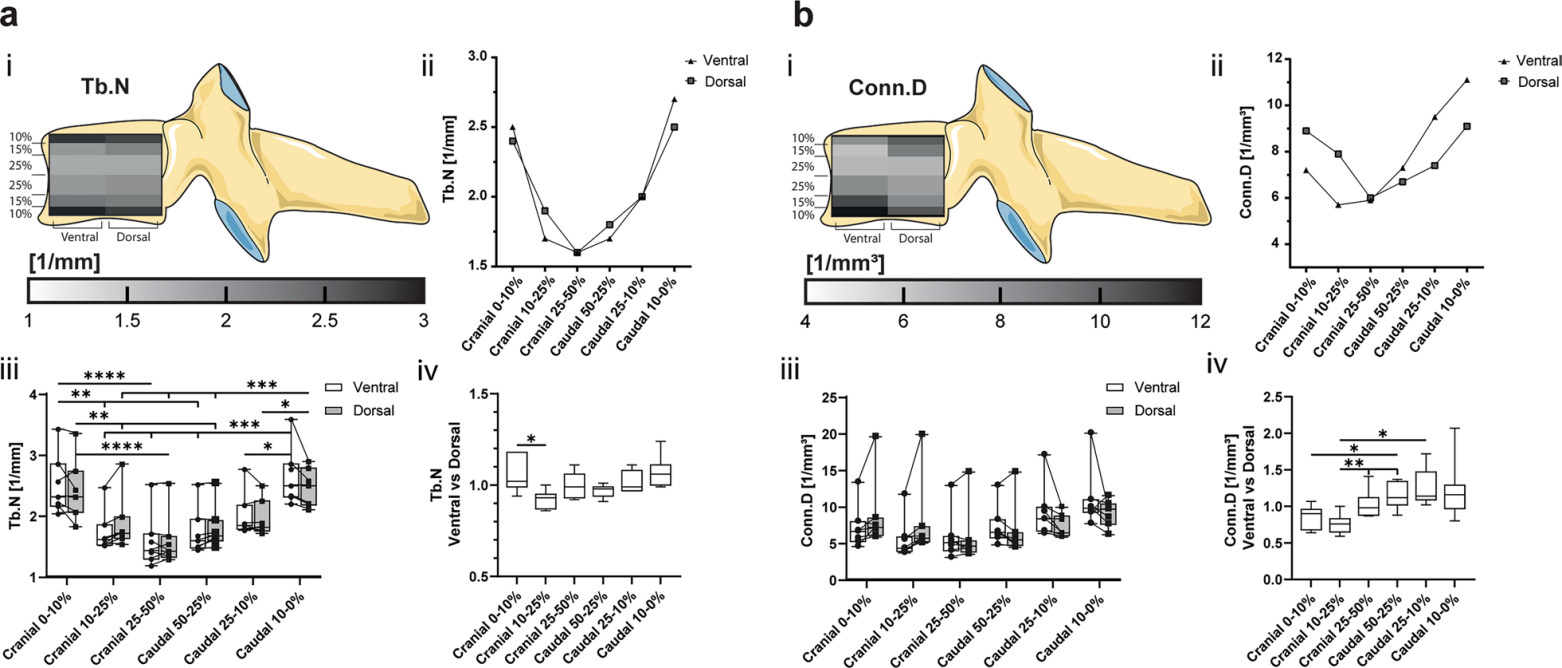
**a** Trabecular number (Tb.N) and **b** connectivity density (Conn.D) are represented in (i) a heatmap corresponding to their cranial–caudal and ventral–dorsal location, (ii) a scatter plot of the mean values of the ventral and dorsal VOI, (iii) a box-and-whisker plot with the individual values for ventral (circle) and dorsal (square) VOI connected by a line (-), and (iv) a box-and-whisker plot of the sample matched ventral–dorsal ratio. (*p < 0.05)

The parameters Tb.Sp and Tb.N vary significantly between the cranial–caudal levels but there is no difference between the ventral and dorsal segments (Figs. 4b, 5a). The lowest Tb.Sp and highest Tb.N are confined to the outer cranial and caudal 10% with values of Tb.Sp from 0.4 ± 0.06 to 0.45 ± 0.07 mm and Tb.N from 2.4 ± 0.5 to 2.7 ± 0.5 mm^−1^. The Tb.Sp values increase towards the centre of the vertebra (0.63 ± 0.14–0.62 ± 0.12 mm) and vice versa the Tb.N decreases towards the centre (1.6 ± 0.4 mm^−1^) (Figs. 4b, 5a).

### Connectivity density increases towards the periphery and is highest ventro-caudally and dorso-cranially

The averaged Conn.D is not significantly changed in any VOI. The highest connectivity is found in the cranial and caudal periphery and decreases towards the core, with the lowest values in the cranial 25–50% (Fig. 5b). The Conn.D displays distinct ventral–dorsal changes in matched comparison. In the cranial 10–25% segment, the Conn.D of the ventral VOI is 76 ± 13% of the dorsal VOI. Interestingly, in the caudal 50–25% and 25–10%, the Conn.D ventrally is higher than in the matched dorsal region and decreases towards the centre regions both ventrally and dorsally (Fig. 5b).

### Segments of the ventral region are more rod-like than their matched dorsal segments

**Fig. 6:**
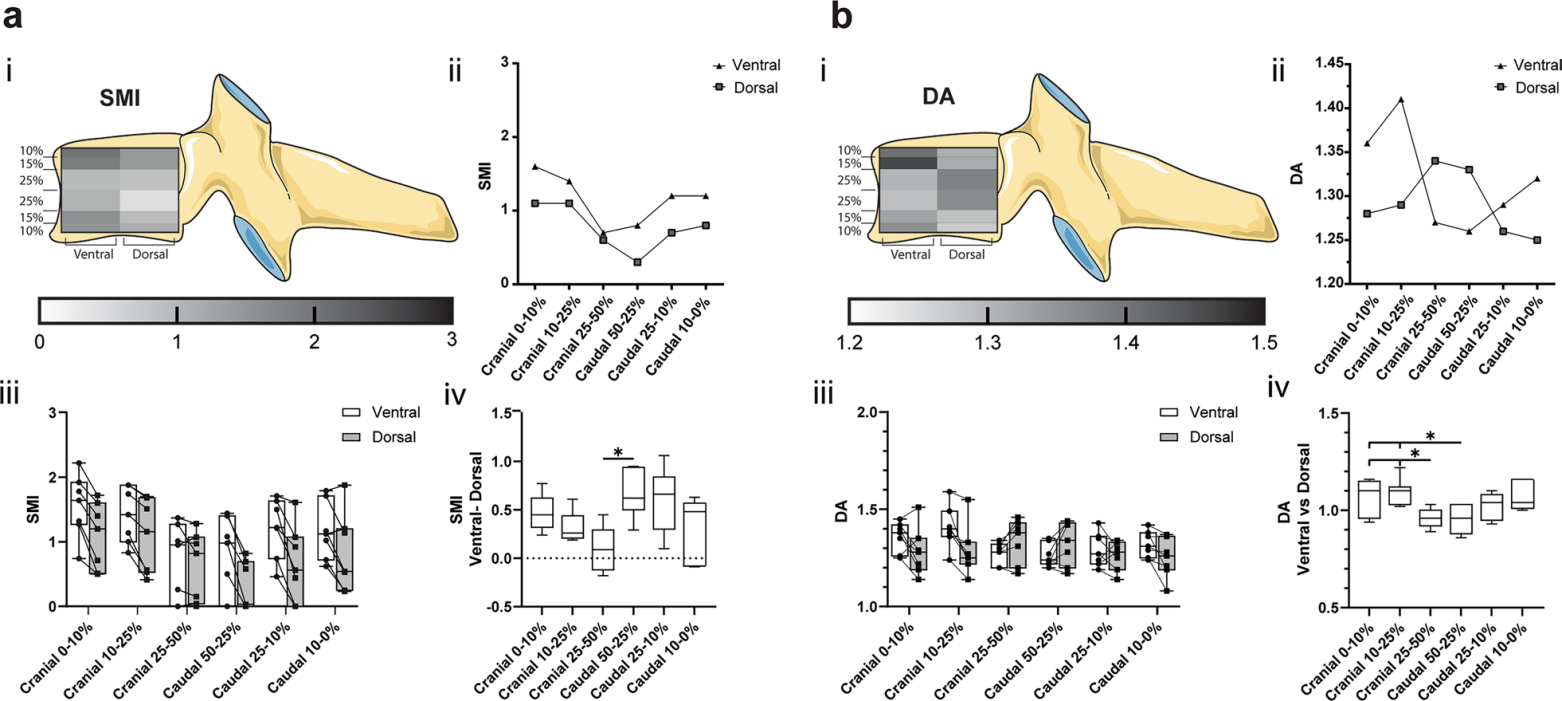
**a** Structure model index (SMI) and **b** degree of anisotropy (DA) are represented in (i) a heatmap corresponding to their cranial–caudal and ventral–dorsal location, (ii) a scatter plot of the mean values of the ventral and dorsal VOI, (iii) a box-and-whisker plot with the individual values for ventral (circle) and dorsal (square) VOI connected by a line (-), and (iv) a box-and-whisker plot of the sample matched ventral–dorsal difference for SMI and ventral–dorsal ratio for DA. (*p < 0.05)

An estimation of the plate-rod trabecular characteristic can be numerically expressed using the structure model index (SMI). Overall, segments of the ventral VOI are more rod-like than their matched dorsal VOIs. The most considerable linear shift in the rod-like direction being a 0.66 ± 0.24 in the caudal 50–25% level and the most minor 0.1 ± 0.22 in the cranial 25–50% (Fig. 6a). Additionally, there is a characteristic pattern of the trabecula where the SMI on the outer cranial levels decreases towards the centre and then increases again as we move caudally away from the core. This is seen in both ventral and dorsal halves.

### Compared to their matched regions, isotropic regions were found ventrally at the extremities and dorsally within the core of the vertebrae

The average DA of all VOI is within the same range from 1.25 to 1.41, without significant differences. The relative change of DA in matched samples shows an isotropic shift from the extremities to the core sections in the ventral region and vice versa in the dorsal region (anisotropic shift) (Fig. 6b).

## Discussion

The present study compared the trabecular architecture of seven C4 vertebrae in twelve predefined layered sections. These were the cranial and caudal 0–10%, 10–15%, and 25–50% in both the ventral and dorsal half of the fourth cervical vertebra. Additionally, the bone mineral density patterns of the endplates of nine C4 vertebrae were analysed with CT-OAM. This study included these two methods to provide a ‘complete’ image of the bone structural parameters of the C4 vertebral body which includes both compact and trabecular bone as both are unavoidably linked during bone functional adaption [14].

Previous studies have looked into the microstructural parameters of the spine in relation to osteoporosis [7], most often focusing on the lumbar spine [15–18]. Regarding the parameters of the cervical spine, reports are sparse. However, mean values of BV/TV and BS/BV are reported as being higher in the C3–C4 region than in the rest of the spine [8, 19, 20]. The denser architecture of the cervical spine might be a result of the high forces which they are exposed to because of their mobility and small size, or, it is hypothesised to be a phylogenetic reminiscence of quadruped gait, where the cervical spine was exposed to much higher stress due to its ‘head-carrying’ position [8].

Of all the parameters reported in this study, Tb.Sp and Tb.N were the only two that displayed significant differences in the averaged values of the VOI. A possible explanation is that the absolute values of the vertebral bone vary as samples come from donors of different ages and sex. This remains a hypothesis and was not investigated further, as this cohort is small and would not provide us with a sound statistical analysis when subdividing it by sex and age. However, distinct and consistent ventral–dorsal modulations were seen in matched sample comparisons, indicating that the relative changes between ventral and dorsal segments are a regular occurrence in the C4 vertebra. Significant cranial–caudal level differences were observed in Tb.Sp and Tb.N. The highest Tb.N was found in the cranial and caudal outer 10%. As Conn.D is also the highest in both these levels, it is not surprising that we also find the lowest Tb.Sp respectively. From here on, an increase in Tb.Sp, as well as a decrease in Tb.N and Conn.D are observed towards the vertebral core. The densely interconnected trabeculae in the superficial levels is most likely related to their endplate adjacent location. As, the trabecular network is designed to sustain vertical compressive sources [21], it is plausible to assume that the superior levels absorb the highest amount of load as they neighbour the endplate and equally dissipate it to the core of the vertebra.

Furthermore, we notice a ventro-dorsal difference in Conn. D and DA. Conn. D is higher at the dorso-cranial and ventro-caudal levels and DA at ventro-cranial level. These are locations directly under the endplates. Previous bone mineralisation densitogram findings effectively mirror this where, across the cervical spine, segment levels reveal a tendential shift in the maxima from the dorsal area to the ventral area specifically in the inferior endplate [6]. From C4 onwards, the highest mineralisation can start to be located dorsally on the superior endplate and ventrally on the inferior endplate because of its transitional position within the cervical vertebrae. These findings are not surprising as the number of connections between trabeculae would be highest in areas of high mineralisation. Similarly, the isotropic properties of the bone in this region also reflects the fact that there is more force applied supero-dorsally and infero-ventrally. CT-OAM is a valuable method of visualisation of the subchondral bone mineralisation. It can be applied to living individuals as it utilises regular CT scans and reflects the long-term chronic loading conditions of the specific bone structure in question [14]. As is the case here, CT-OAM would suffice in reflecting the force transmission and chronic loading conditions within the C4 vertebrae, however, the addition of µCT adds value to a microstructural trabecular level for the analysis of bone quality for surgical instrumentation insertion and durability.

**Fig. 7:**
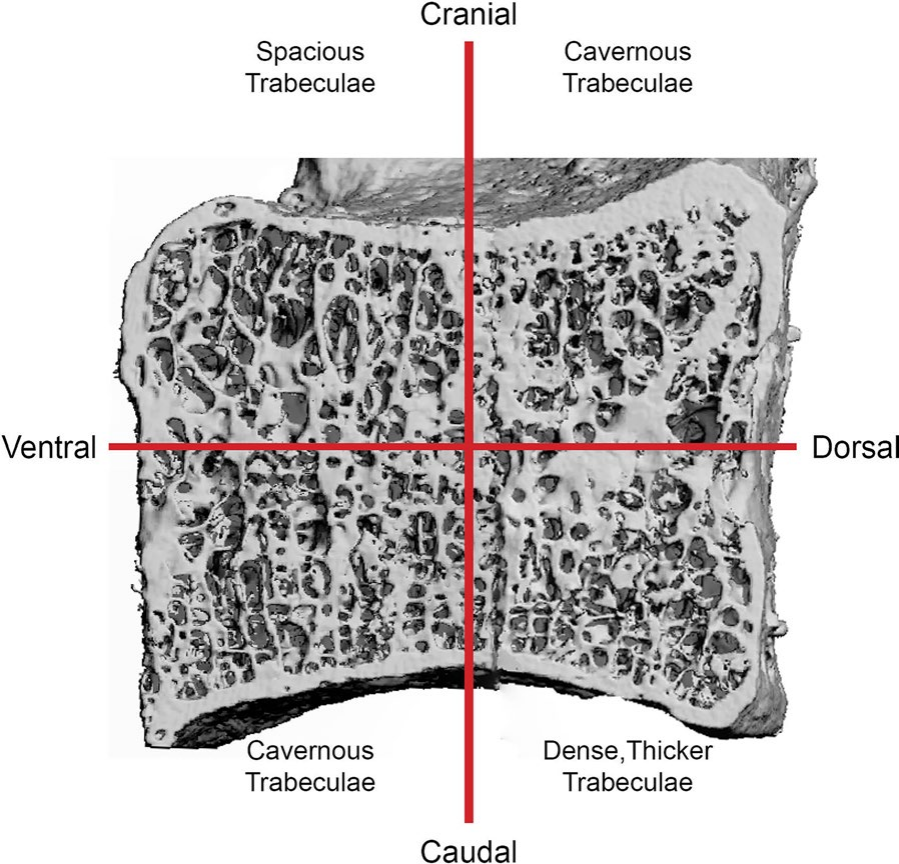
3D µCT reconstruction of a sagittal cut C4 vertebra split into four quadrants corresponding to the cranial–caudal and ventral–dorsal axis. Region-specific descriptions of the trabecular substructure are detailed in the respective quadrant

The consistent ventral to dorsal changes were predominantly observed in the BV/TV, BS/BV, and SMI overall values, as well as in the Tb.Th in the three caudal levels. To simplify here is a summary of each quartered region pertaining to the vertebra's ventral–cranial, dorsal–cranial, ventral–caudal, and dorsal–caudal sections (Fig. 7). The ventral quarters (or half) display lower BV/TV, respectively higher BS/BV and SMI than their sample paired dorsal quarters. The ventral–cranial quarter shows the lowest BV/TV and the highest BS/BV and SMI. In addition, this quarter also presents high Tb.Sp. Collectively, this describes spacious cancellous bone with rod-like trabeculae. In contrast, the dorsal–caudal quarter exhibits the highest BV/TV and Tb.Th and the lowest BS/BV and SMI. These parameters illustrate thicker, denser, and more plate-like trabecula. The dorsal–cranial and ventral–caudal quarters are relatively similar and represent intermediate characteristics of the ventral–cranial and dorsal–caudal regions, characterising the cancellous bone as cavernous with trabeculae with rod-plate-like intermediary features. However, the usage of SMI should now be interrogated in light of recent research [22].

To summarize, the dorsal quadrants are "stronger" in terms of bone density and architectural properties than their ventral counterparts. Moreover, the caudal segments are "stronger" than their adjacent cranial segments in the same regard. This suggests that the strongest part is the caudal dorsal part, and the weakest is the cranial ventral one. When considering the bone functional adaptation phenomenon, these findings are legitimate as the “healthy” cervical spine is, by nature, in a physiological lordosis, which leads the resulting force of the joint to be positioned dorsally. The predominant load exposure in this joint area leads to higher mineralisation and trabecular adaptation than the less strained ventral volume. Regarding implant placement, our data suggest the recommendable should be dorsal, and screws should be optimally anchored in the dorsal–caudal region.

Comprehension of the cancellous bone architecture improves the prediction of biomechanical properties [23]. Furthermore, laboratory failure testing has shown that trabecular structural parameters significantly impact the stability of orthopaedic implants [24–26]. In this regard, our data is invaluable for spinal surgeons to optimise or even develop new implant procedures. The effects of metabolic skeletal disorders on the relationship of the sub-architecture of cancellous bone are hard to predict.

The main limitation of our study is that we could only evaluate the trabecular architecture of the C4 vertebra. As the middle cervical vertebra, it can be assumed that the other regular cervical vertebrae C3 and C5–C7 have similar architectural characteristics due to a comparable loading profile. Although it is anticipated that the values are of different magnitude, it can be expected that intravertebral ratios resemble those of the C4.

## Conclusion

CT-OAM and µCT have shown the relationship between the trabecular and compact bone within the C4 vertebrae which can be visualised in vivo. These methods also demonstrate the interdependence of compact and trabecular bone in response to long-term loading conditions. Highest bone mineralisation is located dorsally. If one assumes that a decrease in bone mass affects the cancellous bone homogenously, then the most stable region remains the dorsal–caudal region. Overall, our data suggest the recommendable placement of orthopaedic implants in the fourth cervical vertebra, such as fusion cages, should be positioned dorsally with screws optimally anchored in the dorsal–caudal region.

## Supplementary Information

**Additional file 1: Figure S1.** Comparison of cancellous bone parameters. a) bone volume fraction (BV/TV), b) bone surface density (BS/BV), c) trabecular thickness (Tb.Th), d) trabecular separation (Tb.Sp), e) trabecular number per volume (Tb.N), f) connectivity density (Conn.D), g) structure model index (SMI), and h) degree of anisotropy (DA) between right (black circle) and left (grey square) ventral and dorsal volumes of interest.

**Additional file 2: Figure S2.** Tables of the results of all bone parameters. Summary tables of all trabecular architecture parameter results with standard deviations and 95% confidence intervals.

## Abbreviations

BS/BV: Bone surface density
BV/TV: Bone volume fraction
Conn.D: Connectivity density
CT-OAM: Computed tomography osteoabsorptiometry
DA: Degree of anisotropy
µCT: Micro-computed tomography
SMI: Structure model index
Tb.N: Trabecular number per volume
Tb.Th: Trabecular thickness
Tb.Sp: Trabecular separation
VOI: Volume of interest
