# Supplementary material for: The quantification of 3D-trabecular architecture of the fourth cervical vertebra using CT osteoabsorptiometry and micro-CT
Source: J Orthop Surg Res. 2023 Apr 12;18:297. doi: 10.1186/s13018-023-03760-2 (PMC10100115; doi:10.1186/s13018-023-03760-2)
Supplement: Supplementary file 2 — Additional file 2: Figure S2. Tables of the results of all bone parameters. Summary tables of all trabecular architecture parameter results with standard deviations and 95% confidence intervals. [file 13018_2023_3760_MOESM2_ESM.docx]

**Supplemental Figure 2**

*CI: 95% Confidence Interval, SD: Standard Deviation*

|  | BV/TV (%) | | | | | | | | |
| --- | --- | --- | --- | --- | --- | --- | --- | --- | --- |
|  | Ventral Mean | SD | CI | Dorsal Mean | SD | CI | Ventral vs Dorsal | SD |  |
| Cranial 0-10% | 21.6 | 8.1 | 0.14-0.29 | 25.9 | 8.7 | 0.18-0.34 | 0.84 | 0.08 |  |
| Cranial 10-25% | 20.9 | 7.6 | 0.14-0.28 | 25.2 | 8.1 | 0.18-0.33 | 0.82 | 0.08 |  |
| Cranial 25-50% | 25.8 | 10.4 | 0.16-0.35 | 27.1 | 10.1 | 0.18-0.36 | 0.95 | 0.11 |  |
| Caudal 50-25% | 26.7 | 10.1 | 0.17-0.36 | 33.0 | 8.6 | 0.25-0.41 | 0.79 | 0.10 |  |
| Caudal 25-10% | 23.8 | 6.0 | 0.18-0.29 | 29.8 | 7.4 | 0.23-0.37 | 0.81 | 0.10 |  |
| Caudal 10-0% | 25.4 | 7.1 | 0.19-0.32 | 28.1 | 5.6 | 0.23-0.33 | 0.91 | 0.20 |  |

|  | BS/BV (1/mm) | | | | | | | | |
| --- | --- | --- | --- | --- | --- | --- | --- | --- | --- |
|  | Ventral Mean | SD | CI | Dorsal Mean | SD | CI | Ventral vs Dorsal | SD |  |
| Cranial 0-10% | 15.2 | 2.2 | 13.1-17.2 | 14.3 | 2.1 | 12.3-16.2 | 1.06 | 0.09 |  |
| Cranial 10-25% | 14.3 | 1.8 | 12.6-16.0 | 13.7 | 1.9 | 12.0-15.4 | 1.04 | 0.07 |  |
| Cranial 25-50% | 12.8 | 1.9 | 11.0-14.6 | 12.3 | 2.0 | 10.5-14.2 | 1.04 | 0.08 |  |
| Caudal 50-25% | 13.6 | 2.4 | 11.4-15.7 | 11.1 | 2.0 | 9.3-12.9 | 1.23 | 0.14 |  |
| Caudal 25-10% | 15.4 | 1.8 | 13.8-17.1 | 12.6 | 2.8 | 10.1-15.2 | 1.24 | 0.13 |  |
| Caudal 10-0% | 15.5 | 2.2 | 13.5-17.6 | 13.9 | 3.0 | 11.1-16.7 | 1.13 | 0.14 |  |

|  | Tb.Th (mm) | | | | | | | | |
| --- | --- | --- | --- | --- | --- | --- | --- | --- | --- |
|  | Ventral Mean | SD | CI | Dorsal Mean | SD | CI | Ventral vs Dorsal | SD |  |
| Cranial 0-10% | 0.16 | 0.03 | 0.13-0.19 | 0.16 | 0.02 | 0.14-0.19 | 0.97 | 0.08 |  |
| Cranial 10-25% | 0.17 | 0.03 | 0.14-0.19 | 0.17 | 0.03 | 0.15-0.20 | 0.98 | 0.07 |  |
| Cranial 25-50% | 0.18 | 0.04 | 0.15-0.22 | 0.19 | 0.04 | 0.16-0.22 | 0.97 | 0.07 |  |
| Caudal 50-25% | 0.18 | 0.04 | 0.14-0.21 | 0.21 | 0.04 | 0.18-0.25 | 0.84 | 0.11 |  |
| Caudal 25-10% | 0.16 | 0.02 | 0.13-0.18 | 0.18 | 0.04 | 0.15-0.22 | 0.85 | 0.08 |  |
| Caudal 10-0% | 0.14 | 0.02 | 0.13-0.16 | 0.17 | 0.03 | 0.14-0.19 | 0.89 | 0.11 |  |

|  | Tb.Sp (mm) | | | | | | | |
| --- | --- | --- | --- | --- | --- | --- | --- | --- |
|  | Ventral Mean | SD | CI | Dorsal Mean | SD | CI | Ventral vs Dorsal | SD |
| Cranial 0-10% | 0.45 | 0.07 | 0.38-0.52 | 0.44 | 0.08 | 0.37-0.51 | 1.02 | 0.08 |
| Cranial 10-25% | 0.58 | 0.09 | 0.50-0.66 | 0.52 | 0.10 | 0.44-0.61 | 1.12 | 0.07 |
| Cranial 25-50% | 0.63 | 0.14 | 0.50-0.77 | 0.62 | 0.12 | 0.51-0.74 | 1.02 | 0.07 |
| Caudal 50-25% | 0.56 | 0.10 | 0.46-0.65 | 0.53 | 0.08 | 0.46-0.61 | 1.04 | 0.07 |
| Caudal 25-10% | 0.49 | 0.07 | 0.43-0.56 | 0.49 | 0.06 | 0.44-0.55 | 1.00 | 0.07 |
| Caudal 10-0% | 0.40 | 0.06 | 0.35-0.46 | 0.42 | 0.04 | 0.38-0.45 | 0.97 | 0.11 |

|  | Tb.N (1/mm) | | | | | | | | |
| --- | --- | --- | --- | --- | --- | --- | --- | --- | --- |
|  | Ventral Mean | SD | CI | Dorsal Mean | SD | CI | Ventral vs Dorsal | SD |  |
| Cranial 0-10% | 2.5 | 0.5 | 2.06-2.97 | 2.4 | 0.5 | 1.93-2.88 | 1.05 | 0.09 |  |
| Cranial 10-25% | 1.7 | 0.3 | 1.42-2.06 | 1.9 | 0.5 | 1.47-2.30 | 0.93 | 0.05 |  |
| Cranial 25-50% | 1.6 | 0.4 | 1.18-2.00 | 1.6 | 0.4 | 1.20-2.00 | 1.00 | 0.07 |  |
| Caudal 50-25% | 1.7 | 0.4 | 1.39-2.10 | 1.8 | 0.4 | 1.47-2.13 | 0.97 | 0.03 |  |
| Caudal 25-10% | 2.0 | 0.4 | 1.67-2.34 | 2.0 | 0.3 | 1.69-2.24 | 1.02 | 0.06 |  |
| Caudal 10-0% | 2.7 | 0.5 | 2.21-3.10 | 2.5 | 0.3 | 2.18-2.76 | 1.07 | 0.08 |  |

|  | Conn.D (1/mm^3^) | | | | | | | | |
| --- | --- | --- | --- | --- | --- | --- | --- | --- | --- |
|  | Ventral Mean | SD | CI | Dorsal Mean | SD | CI | Ventral vs Dorsal | SD |  |
| Cranial 0-10% | 7.2 | 2.9 | 4.5-10.0 | 8.9 | 4.8 | 4.4-13.3 | 0.85 | 0.16 |  |
| Cranial 10-25% | 5.7 | 2.8 | 3.1-8.3 | 7.9 | 5.4 | 2.9-12.8 | 0.76 | 0.13 |  |
| Cranial 25-50% | 5.9 | 3.2 | 2.9-8.9 | 6.0 | 3.9 | 2.3-9.6 | 1.03 | 0.19 |  |
| Caudal 50-25% | 7.3 | 2.7 | 4.9-9.8 | 6.7 | 3.6 | 3.3-10.1 | 1.16 | 0.18 |  |
| Caudal 25-10% | 9.5 | 3.6 | 6.1-12.8 | 7.4 | 1.6 | 6.0-8.9 | 1.26 | 0.25 |  |
| Caudal 10-0% | 11.1 | 4.1 | 7.3-14.9 | 9.1 | 1.8 | 7.5-10.8 | 1.23 | 0.41 |  |

|  | SMI | | | | | | | |
| --- | --- | --- | --- | --- | --- | --- | --- | --- |
|  | Ventral Mean | SD | CI | Dorsal Mean | SD | CI | Ventral - Dorsal | SD |
| Cranial 0-10% | 1.6 | 0.5 | 1.1-2.0 | 1.1 | 0.5 | 0.6-1.6 | 0.47 | 0.18 |
| Cranial 10-25% | 1.4 | 0.4 | 1.0-1.8 | 1.1 | 0.6 | 0.6-1.6 | 0.34 | 0.16 |
| Cranial 25-50% | 0.7 | 0.6 | 0.1-1.2 | 0.6 | 0.5 | 0.1-1.1 | 0.10 | 0.22 |
| Caudal 50-25% | 0.8 | 0.6 | 0.1-1.3 | 0.3 | 0.4 | 0.0-0.7 | 0.66 | 0.24 |
| Caudal 25-10% | 1.2 | 0.5 | 0.8-1.6 | 0.7 | 0.6 | 0.1-1.2 | 0.61 | 0.32 |
| Caudal 10-0% | 1.2 | 0.4 | 0.8-1.6 | 0.8 | 0.6 | 0.3-1.4 | 0.34 | 0.30 |

|  | DA | | | | | | | |
| --- | --- | --- | --- | --- | --- | --- | --- | --- |
|  | Ventral Mean | SD | CI | Dorsal Mean | SD | CI | Ventral vs Dorsal | SD |
| Cranial 0-10% | 1.36 | 0.08 | 1.29-1.43 | 1.28 | 0.12 | 1.17-1.40 | 1.07 | 0.09 |
| Cranial 10-25% | 1.41 | 0.11 | 1.31-1.51 | 1.29 | 0.13 | 1.17-1.41 | 1.10 | 0.07 |
| Cranial 25-50% | 1.27 | 0.06 | 1.22-1.33 | 1.34 | 0.11 | 1.23-1.44 | 0.96 | 0.05 |
| Caudal 50-25% | 1.26 | 0.06 | 1.21-1.32 | 1.33 | 0.11 | 1.22-1.43 | 0.96 | 0.07 |
| Caudal 25-10% | 1.29 | 0.09 | 1.21-1.38 | 1.26 | 0.07 | 1.19-1.33 | 1.03 | 0.07 |
| Caudal 10-0% | 1.32 | 0.07 | 1.26-1.38 | 1.25 | 0.10 | 1.16-1.34 | 1.06 | 0.07 |
